# Emulating proton transfer reactions in the pseudo-protic ionic liquid 1-methylimidazolium acetate

**DOI:** 10.1039/d2cp00643j

**Published:** 2022-04-07

**Authors:** Richard Jacobi, Florian Joerg, Othmar Steinhauser, Christian Schröder

**Affiliations:** Institute of Theoretical Chemistry, Faculty of Chemistry, University of Vienna Währinger Straße 17 1090 Vienna Austria; Vienna Doctoral School in Chemistry (DoSChem), University of Vienna Währinger Straße 42 1090 Vienna Austria; Department of Computational Biological Chemistry, Faculty of Chemistry, University of Vienna Währinger Straße 17 1090 Vienna Austria christian.schroeder@univie.ac.at +43 1 4277 52711

## Abstract

Proton transfer reactions can enhance conductivity in protic ionic liquids. However, several proton reactions are possible in a multicomponent system of charged and neutral species, resulting in a complex reaction network. Probabilities and equilibrium concentrations of the involved species are modeled by the combination of reducible Markov chains and quantum-mechanical calculations.

## Abstract 2

Proton transfer reactions can enhance conductivity in protic ionic liquids.

## Graphical abstract

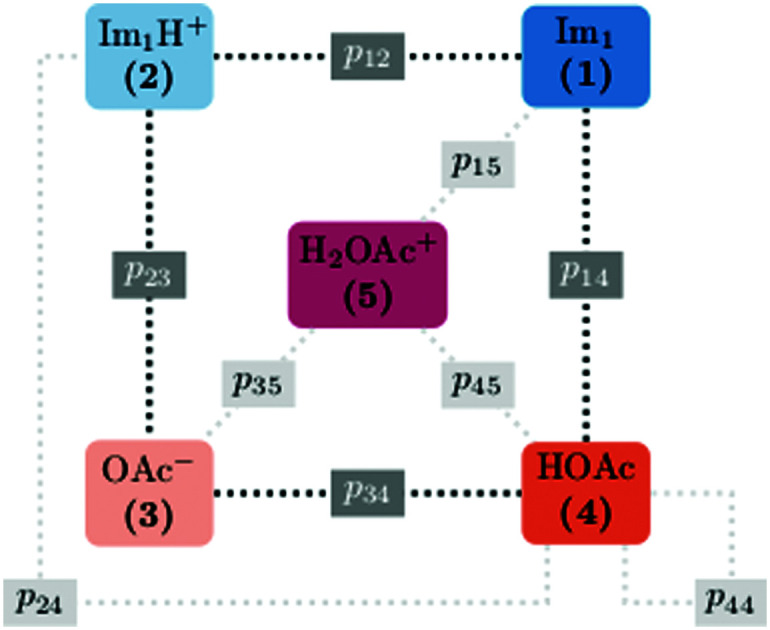

## 1 Introduction

Ionic liquids play a promising role in future battery and fuel cell generations and can contribute positively to the current challenges of alternative and sustainable energy usage.^1–3^ Of particular interest are protic ionic liquids (PILs), a subclass of ionic liquids composed of a Brønsted acid and Brønsted base. These liquids can transfer a proton and form hydrogen-bonded networks resulting in high conductivities.^4,5^

**Fig. 1:**
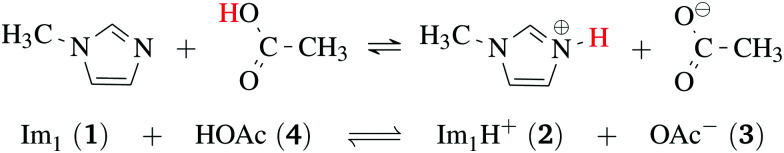
Formation of 1-methylimidazolium acetate [Im_1_H]OAc. Several experiments indicate that the equilibrium is on the left side.^7,8,10^ Strictly speaking, this fact disqualifies the mixture to be classified as an ionic liquid. However, this pseudo-PIL shows a significant conductivity arguing for significant concentration of molecules on the right hand side.

*Ab initio* molecular dynamics simulations are a good tool for examining proton transfer reactions, as bond breaking and formation occur naturally. In a combined NMR, IR, and *ab initio* study MD study on the proton transfer between carboxylates and pyridines, the proton transfer pathway was studied.^6^ In a recent AIMD study, Kirchner and co-workers^7^ found evidence for a Grotthuss diffusion mechanism in the 1-methylimidazolium acetate [Im_1_H]OAc system depicted in Fig. 1, which may explain the high experimental conductivity of this PIL.^8,9^

**Fig. 2:**
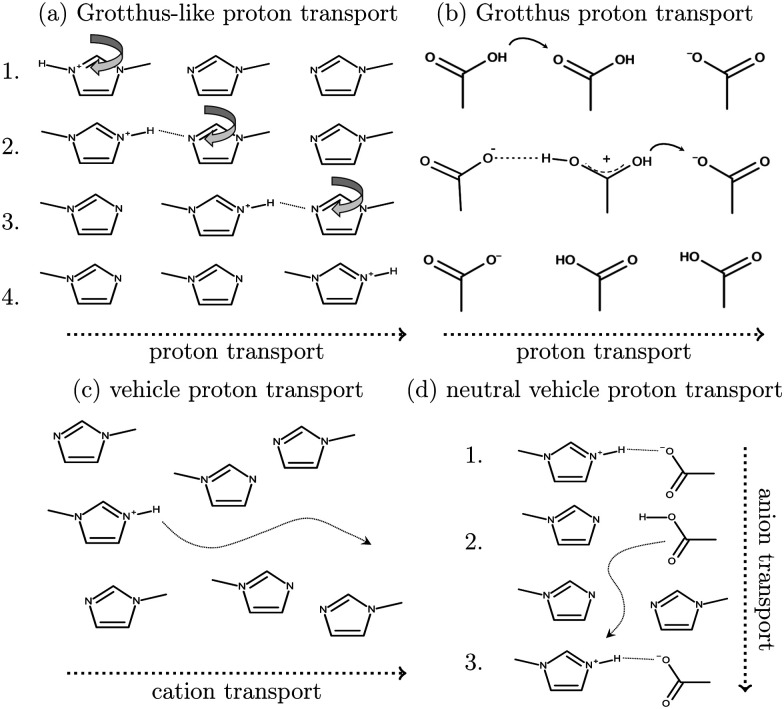
Various charge transport mechanisms for the conductivity in protic ionic liquids. Grotthuss-like proton transport (a) is also possible using a rotating acetic acid molecule. A direct Grotthuss-mechanism can be realized *via* protonated acetic acid molecules (b). Vehicle transport can be realized *via* cations (c) or neutral acetic acid (d).

As shown in Fig. 2a, Grotthuss-like proton transfer can be realized *via* rotating 1-methylimidazolium cations or acetic acid molecules.^9^ Strictly speaking, a Grotthuss mechanism requires the transfer of one proton to the molecule and the release of another proton at the same molecule. This can only be achieved *via* protonated acetic acid molecules H_2_OAc^+^ which were also found in the study of Kirchner and co-workers.^7^ In addition, proton transport *via* a vehicle mechanism is also possible (Fig. 2c and d). Intuitively, 1-methylimidazolium is the proton carrier in this scenario but was excluded by Umebayashi and co-workers.^9^ However, as neutral molecules diffuse much faster than their charged counterparts, neutral vehicle transport may also be relevant. In this case, a proton is transferred from 1-methylimidazolium to an acetate molecule. This reaction is favored compared to the back reaction (see Fig. 1). The neutral molecule diffuses through the liquid. At some point, the back reaction may take place. As a result, from step 1 to step 3, an acetate molecule moved with less electrostatic friction.

We would like to evaluate which of these proposed mechanisms is relevant for the conductivity in the PIL [Im_1_H]OAc. However, *ab initio* simulations are restricted to system sizes of a few ion pairs and simulation times of dozens of picoseconds, making it impossible to evaluate essential properties such as conductivity. In contrast, molecular dynamics simulations easily handle hundreds of ion pairs and simulation periods of several dozens of nanoseconds. While classical molecular dynamics simulations fail to predict dynamic properties accurately,^11–13^ polarizable force fields, implemented usually *via* point inducible dipoles,^14–16^ fluctuating charges^17,18^ or Drude oscillators,^19^ were established to improve accuracy, especially for dynamical properties.

As a first step for the polarizable molecular dynamics simulations involving proton transfer reactions, we have to determine the probabilities for protonation of 1-methylimidazole, acetate, and acetic acid as well as for the deprotonation of 1-methylimidazolium and (protonated) acetic acid. Consequently, we performed quantum-mechanical scans of the (de-)protonations using a polarizable continuum model to model the dielectric background of the liquid phase. However, we also want to make sure, that the ratio of components in Fig. 1 follows the experimental density data,^10^*i.e.* roughly 30% ionic and 70% neutral molecules.^7,10^ For that reason, we apply reducible Markov chain models to scan the complete “phase space” of possible reaction probability combinations and the resulting equilibrium concentrations.

## 2 Theory and methods

### 2.1 The network of reactions

**Fig. 3:**
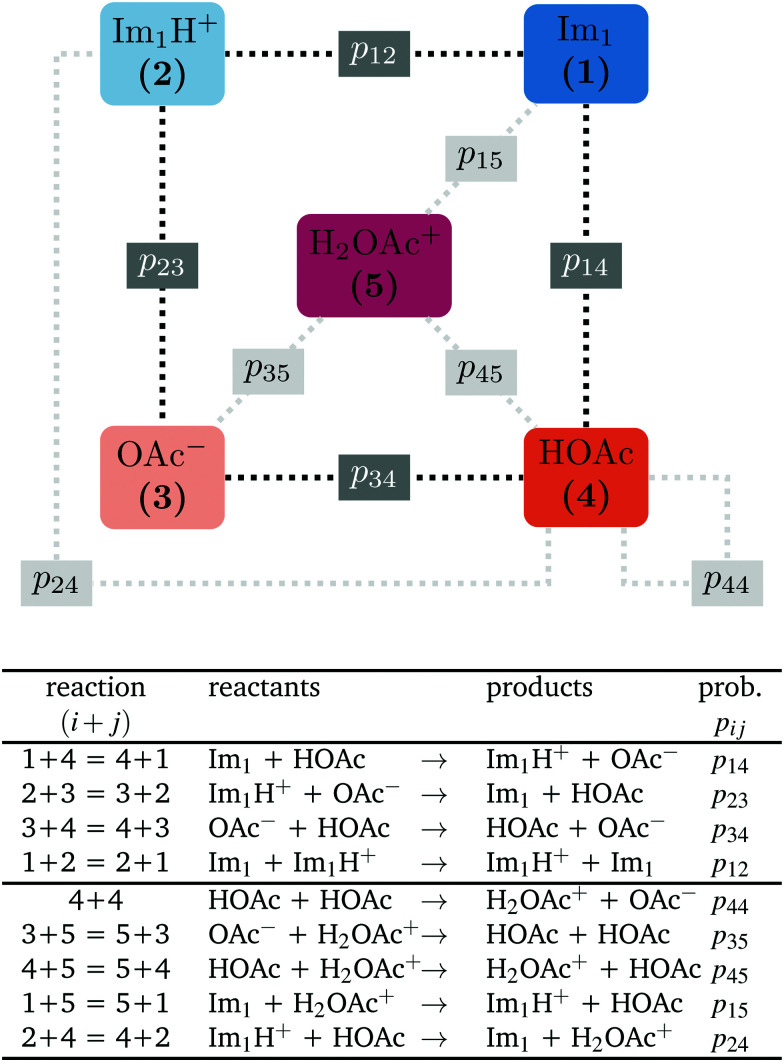
The following proton transfer reactions (*i* + *j*) are considered in this work. The first four reactions apply to the simple protonation scheme (dark gray boxes). In the advanced protonation scheme, five additional reactions are considered (light gray boxes). Please note that the numbers for the reaction (*i* + *j*) denote the reacting species. Consequently, the sequence does not play a role and (4 + 1) is not the back reaction of (1 + 4).

In contrast to the rudimentary protonation scheme reported in ref. 9, we distinguish between a simple and an advanced protonation scheme (see Fig. 3) in this work: The simple protonation scheme involves proton transfer reactions between the species 1-methylimidazole (Im_1_, 1), 1-methylimidazolium (Im_1_H^+^, 2), acetate (OAc^−^, 3), and acetic acid (HOAc, 4). The advanced protonation scheme additionally includes the protonated acetic acid (H_2_OAc^+^, 5), which Kirchner and co-workers detected in AIMD simulations.^7^

The simple protonation scheme contains the equilibrium displayed in Fig. 1 modeled by the forward reaction (1+4) and backward reaction (2+3) in Fig. 3. Additionally, the proton exchange between acetic acid and acetate (reaction (3+4)) as well as between 1-methylimidazole and 1-methylimidazolium (reaction (1+2)) may occur. However, the last two reactions do not change the concentration of the species but may be necessary for the proton transfer mechanism (see Fig. 2). The inclusion of the protonated acetic acid H_2_OAc^+^ in the advanced protonation scheme adds five additional reactions for possible proton transfers. In particular, the reactions (4+4), (3+5), and (4+5) are essential for the Grotthuss mechanism in Fig. 2b. The reactions (1+5) and (2+4) involve the proton transfer between a carboxylic and an imidazole-based compound. Each of these reactions (*i*+*j*) has a probability *p*_*ij*_ to take place, assuming that the reacting pair already exists. However, these reactions occur simultaneously with different probabilities and mole fractions *x*_*i*_ of the reacting partners. The complex network emerging from these reactions will result in an equilibrium distribution of the participating species.

All combinations of species 1 to 5 not mentioned in Fig. 3 (and not connect *via* a dashed line) do not react with each other. Consequently, their reaction probabilities *p*_*ij*_ are zero:

1 *p*_11_ = *p*_13_ = *p*_31_ = *p*_22_ = *p*_25_ = *p*_52_ = *p*_33_ = *p*_55_= 0

### 2.2 Markov chain models of the simple and advanced protonation scheme

Markov chains are stochastic models predicting equilibrium distributions of the reacting species. Each molecular species *i* is a Markov state in the present case. Its population changes due to the (de-)protonation reactions in Fig. 3. For example, the protonation of Im_1_ during the reaction (1+4) yields Im_1_H^+^. At the same time HOAc is deprotonated and turned into OAc^−^. Consequently, the mole fractions of *x*_1_ and *x*_4_ decrease and *x*_2_ and *x*_3_ increase.

Quite generally, the transition rate 
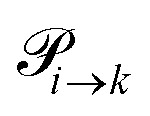
 emerges from the sum of products of the probability to find a suitable reacting partner *x*_*j*_ and the probability *p*_*ij*_ that the reaction (*i*+*j*) actually takes places:2
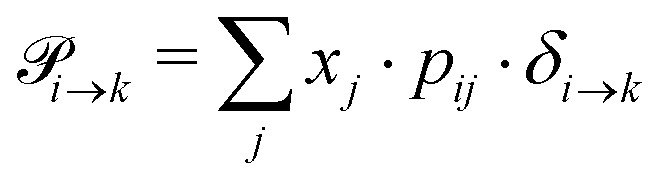
The index *i* denotes the species before the (de-)protonation. However, *j* is the reaction partner and not necessarily the (de-)protonated form *k* of species *i*. The transition function *δ*_*i*→*k*_ ensures that only those *p*_*ij*_ which leads to the conversion of *i* to *k* should be considered in this summation.

**Fig. 4:**
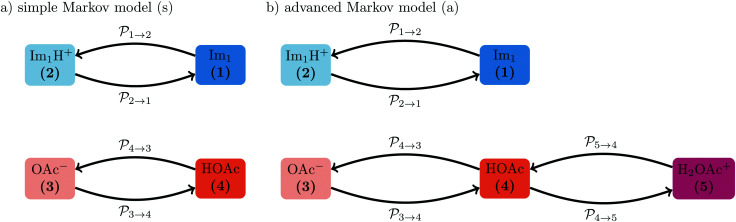
The simple (s) and advanced (a) Markov chain model are both reducible as not every state can be reached from a starting state in a multistep process. The transition rate 
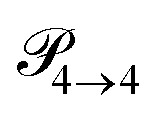
 is not displayed as it does not change the mole fraction *x*_4_.

The transition rates 
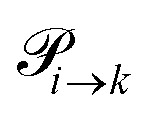
 are not directly visible in Fig. 3 as only the reacting partners *i* and *j* are connected *via* dashed lines. Consequently, Fig. 3 is not a classical picture of a Markov chain which is given instead in Fig. 4. In the simple protonation scheme, all four species have exactly two channels to react (labeled by the dark gray boxes in Fig. 3). Therefore, the sum in eqn (2) consists of two contributions of each 
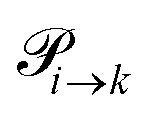
. This changes for the advanced protonation scheme. All species except for HOAc now have three reaction pathways (see Fig. 3). However, HOAc now has five reaction pathways {(1+4),(3+4),(4+4),(4+5),(2+4)} and seems to be the most important molecule at first sight. This leads to a little bit more complex transition rates in the advanced model compared to 
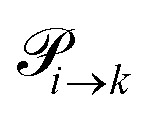
 of the simple model due to the additional contributions (light gray boxes in Fig. 3).

The probabilities 
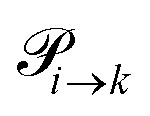
 to get from one state *i* to another one *k* are gathered in a transition matrix 
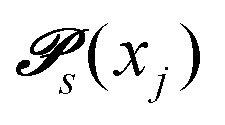
 (simple protonation)3
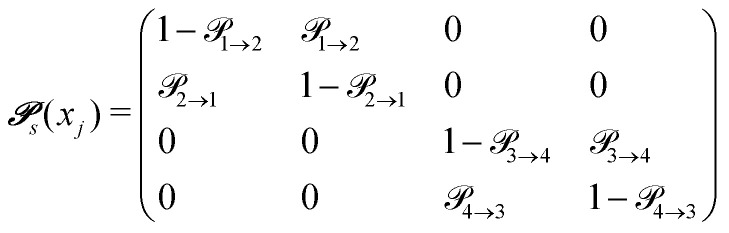
and 
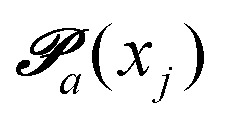
 (advanced protonation)4
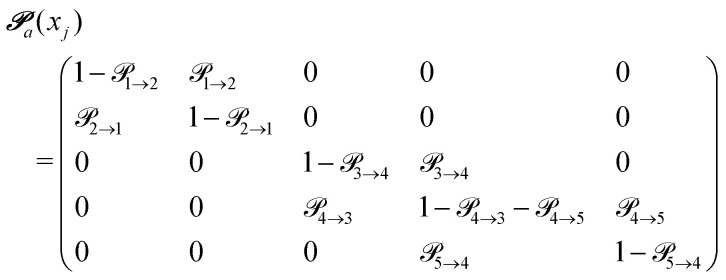
Due to the additional protonated acetic acid H_2_OAc^+^5 the size of the transition matrix 
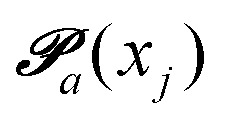
 increased to 5 × 5. Both transition matrices are stochastic, *i.e.* the sum of each row yields unity. That does not apply to each column. Furthermore, the mole fractions *x*_*j*_ of the species enter the transition matrix to find the corresponding partners (see eqn (2)).

Given an initial distribution of states *x⃑*(0) = (*x*_1_(0),…,*x*_5_(0)) at time *t* = 0, the population after *t* time steps can be obtained by applying the following equation *t* times:5
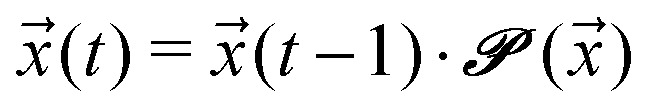


The sum of all mole fractions is normalized to unity at all times, *i.e.*
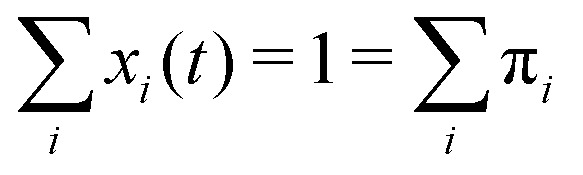
. For *t* → ∞, the state populations reach a steady state:6
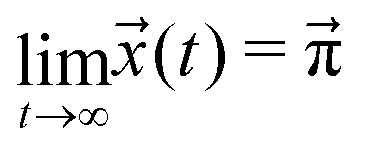
7
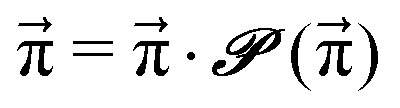


These Markov models serve as a test system for determining meaningful reaction probabilities *p*_*ij*_ resulting in the correct (=experimental) equilibrium mole fractions 
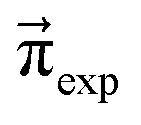
. Knowing the final state distribution is our significant advantage over the studies of Gillespie using a Monte Carlo approach for the stochastic formulation of chemical kinetics.^20^

We scan all probabilities *p*_*ij*_ from 0% to 100% in steps of 5% and determine the steady-state concentrations π by computing *x*(*t* = 1000) iteratively from eqn (5) as this number corresponds to the number of protonation events planned during the polarizable, proton transfer MD simulations. In order to further increase computational efficiency, we start at the experimental mole fractions 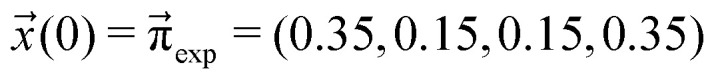 for the simple protonation scheme corresponding to 30% ionic and 70% neutral molecules. Our initial guess for the advanced protonation scheme is 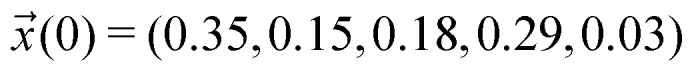. Here, we overestimated the initial concentration of the protonated acetic acid 5 on purpose to allow for corresponding reaction involving this species. Starting with a more realistic concentration near zero would immediately prohibit the reactions {(3+5),(4+5),(1+5)} to take place.

Since we are more or less close to the expected equilibrium, the number of iterations *t* to reach the final computational state distribution 
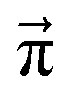
 may be lower. Therefore, we check the norm |*x⃑*(*t*) − *x⃑*(*t* − 1)| of the change of the mole fraction at each iteration and stop the loop if this norm is less than 10^−5^ to safe computational time. This procedure is computationally at least one order of magnitude faster than the standard eigenvalue method. Usually, the steady-state population 
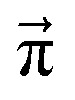
 is determined *via* the eigenvector of the highest eigenvalue 1.^21^

However, not all sets of *p*_*ij*_ necessarily stay close to *x⃑*(0). For example, if *p*_14_ is significantly larger than *p*_23_, the computational equilibrium characterized by 
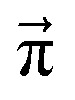
 will shift to the right hand side of Fig. 1 and hence will be quite different to 
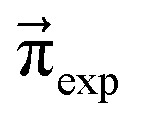
. A set of *p*_*ij*_ is accepted, if it fulfills the following conditions:

• Charge neutrality.

• Balanced species.

• Ratio of ionic : neutral molecules should be close to 30% : 70%.

Charge neutrality reads in the simple and advanced protonation scheme π_2_ − π_3_ ≃ 0 and π_2_ − π_3_ + π_5_ ≃ 0, respectively. We accept a difference of 0.001 for numerical reasons. As both Markov chain models are reducible, the number of imidazolium-based and carboxylate-based species should be the same. Im_1_ and Im_1_H^+^ cannot react to OAc^−^, HOAc or H_2_OAc^+^, and *vice versa*. Consequently, the balance is checked *via* π_1_ + π_2_ − π_3_ − π_4_ − π_5_ ≃ 0. In the case of the simple protonation scheme, the protonated acetic acid is missing, *i.e.*, π_5_ = 0. Again, we accept a difference of 0.001 for numerical reasons. The last condition requires π_1_ − 0.35 = 0 to yield 30% ionic: 70% neutral molecules. Here, we allow for a deviation of 0.05 for both protonation schemes as this experimental ratio cannot be determined with high precision.

### 2.3 Quantum-mechanical scans

Each of the final valid sets of {*p*_14_,*p*_23_,*p*_34_,*p*_12_,*p*_44_,*p*_35_, *p*_45_,*p*_15_,*p*_24_} may be considered as a point in the “probability space” of meaningful mathematical reaction probabilities. However, almost a billion of these combinations exist for the advanced protonation scheme. In order to restrict this huge phase space to chemical meaningful probabilities, we apply additional quantum-mechanical scans.

All quantum mechanical calculations were performed using the program package Gaussian 16^22^ on the B3LYP/6-311++G(d,p) level of theory. Dispersive effects were corrected empirically using Grimmes model of version D3 with Becke–Johnson damping (D3BJ).^23^ Solvent effects were included using the polarizable continuum model (PCM). The static dielectric constant of the ionic liquid was set to the experimental value of 33.3,^24^ which is also similar to the value reported in the study of Umebayashi and co-workers.^9^

**Fig. 5:**
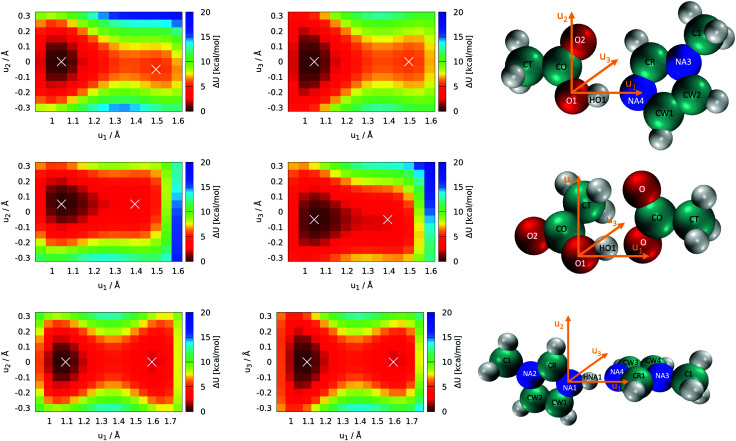
Rigid 2D proton position scans on the optimized structures. Axes *u*_1_, *u*_2_ and *u*_3_ denote the scan directions as visualized also in the molecules. White crosses denote the local minima. The pictures on the right contain the atom names used in the text. **Top panel (1+4),(2+3):** scans of the acetic acid (HOAc, 4)/1-methylimidazole (Im_1_, 1) pair with the protic hydrogen HO1. **Middle panel (3+4):** scans of the acetic acid (HOAc, 4)/acetate (OAc^−^, 3) pair with the protic hydrogen HO1. **Bottom panel (1+2):** scans of the 1-methylimidazole (Im_1_, 1) pair/1-methylimidazolium (Im_1_H^+^, 2) pair with the protic hydrogen HNA1.

Optimization of all five individual molecules, as well as pairs of them, was done. Binding energies were computed as the energy difference between the pair of molecules and the sum of the individual energies. Single-point calculations were performed on the frozen molecules to explore the topology of the potential energy surface with respect to the position of the proton. These scans of the proton's position were performed with all atomic coordinates fixed to the optimized values, and only the position of the proton was manipulated. Thus, these scans are labeled as “rigid” (see Section 3.1). The acidic proton (HO1 and HNA1, respectively, see Fig. 5) was scanned along with two grids with a mesh size of 0.05 Å. One of the grid planes was aligned with the molecular plane, thus including axes *u*_1_ and *u*_2_ as seen in Fig. 5. The second plane was imposed perpendicular to the first one in such a way that the joint straight of the two planes coincides with the direct line between the hetero atoms of acid and base, hence including axes *u*_1_ and *u*_3_ = *u*_2_ × *u*_1_. As these twodimensional scans revealed that the most likely proton transfer happens more or less along a straight line connecting the hydrogen bond donor and acceptor, additional scans using this reaction coordinate were performed.

Starting from the optimized pair structures, relaxed onedimensional scans were performed by increasing the distance between the proton and its parent atom (see Section 3.2). In each step, the geometries were optimized, and the ground state energy was computed. Aside from the freezing of the respective H–X distance (X = N, O) at each step, no further restraints were imposed on the systems. As a result, the geometries can freely adapt to each given H–X distance. An exception is the system HOAc–HOAc, where the second H–O distance was frozen concerning the equilibrium optimized ground state geometry. This assured that OAc^−^ and H_2_OAc^+^ were formed while a proton exchange reaction was prohibited. The step size for the H–X distance was set to 0.025 Å.

## 3 Results and discussion

### 3.1 Rigid twodimensional scans of the reaction partners

The most important reactions of Fig. 3 concern proton transfers between Im_1_, Im_1_H^+^, HOAc and OAc^−^. This generates the need for a decent understanding of these reaction profiles.

The optimal configuration of Im_1_ and HOAc in the top panel of Fig. 5 is about 14.2 kcal mol^−1^ lower in energy (left white *x* of the top heatmap) compared to the sum of both isolated molecules. This energy difference can be regarded as an approximation for the intermolecular binding energy of the two structures. Upon binding, the O1–HO1 distance gets slightly elongated from 0.97 Å in the isolated acetic acid to 1.03 Å, indicating a hydrogen bond interaction. Both molecules align in the same molecular plane, caused by the hydrogen bonding interactions HO1–NA4 and O2–HCR. In the minimum structure, the protic hydrogen is bonded to HOAc. The rigid-geometry scans in the top panel of Fig. 5 exhibit an energy difference of around 5.4 kcal mol^−1^ between the two local minima, which corresponds to hydrogen binding to either HOAc or Im_1_H^+^ (right white *x* in the top heatmap). These two minima of the rigid twodimensional scan are separated by an energy barrier of around 6.0 kcal mol^−1^ (referenced to the global minimum). The scan within the molecular plane, which encompasses *u*_1_ and *u*_2_, exhibits quite a significant asymmetry comparing positive and negative values of *u*_2_. This is most probably caused by a repulsive interaction between the protic hydrogen and the second hydrogen bonding interaction between O2 and HCR. Resulting from this, the local minimum corresponding to Im_1_H^+^ is not on the line directly connecting O1 and NA4, but shifted slightly within the plane, matching a bond angle relaxation at NA4.

In contrast, the perpendicular energy surface, encompassing *u*_1_ and *u*_3_, is notably more symmetric, as there are no significant structural differences above and below the molecular plane. At the same time, this potential energy surface is shallower compared to the in-plane heatmap, indicating less constraint on the proton along this plane. Nevertheless, both scans in the top panel of Fig. 5 indicate that the optimal transition pathway is directly between O1 and NA4, as deviations from the optimal path result in unfavorable energy levels. Consequently, more detailed quantum-mechanical scans may be performed with a onedimensional reaction coordinate along the OH-bond (see Section 3.2).

In addition to the equilibrium displayed in Fig. 1 protonation and deprotonation can also occur between HOAc/OAc^−^ (reaction 3+4) and Im_1_/Im_1_H^+^ (reaction 1+2). The binding energy of 16.7 kcal mol^−1^ between HOAc and OAc^−^ is slightly higher than between HOAc and Im_1_. Accordingly, the HO1–O1 distance of 1.05 Å is more elongated in the HOAc/OAc^−^ pair compared to HOAC/Im_1_. This represents a more intense hydrogen bond, as expected when exchanging nitrogen for the more electronegative oxygen. The second hydrogen bond in the HOAC/OAc^−^ arises between the second O of acetate and one of the HCT atoms of acetic acid (see middle panel of Fig. 5). As a result, both acetate frameworks are not aligned in the same molecular plane but rotated; the dihedral 
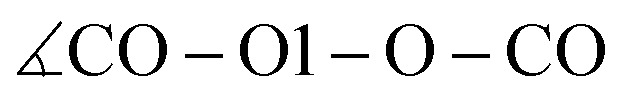
 is 65°.

Generally, for a proton transfer between OAc^−^ and HOAc to take place, no energy difference between the two minimum structures is to be expected. However, the frozen geometry scans in the middle panel of Fig. 5 display an energy difference between the two protonated states of 2.7 kcal mol^−1^, with no energy barrier in between. This energy difference is partly due to the rigidity of the structures, which prohibits any structural relaxation caused by the different intermolecular arrangements of the molecule. Additionally, in the global minimum, the second O atom in OAc^−^ develops a hydrogen bonding interaction to the methyl group of HOAc, as previously mentioned. After the proton transfer, this hydrogen bond still persists, but the reduction in partial charge on the free O in OAc^−^ upon protonation weakens this interaction. This results in a somewhat artificial rise in total energy connected to the proton transfer, which in reality can be overcome by rearrangement of the molecules or coordination to additional molecules in the direct vicinity.

Additionally, both scans are highly asymmetric, which is due to the acetate and acetic acid molecules not aligning within the same plane. In consequence, the scans correspond to the molecular plane or the plane perpendicular to that, respectively, of the acetic acid molecule only but are in turn widely off-plane for the acetate. Although the proton transfer corridor is wider than Im_1_/HOAc, a onedimensional scan along the two active oxygen is still the pathway with the least energy barrier.

Looking at the bottom panel of Fig. 5 the binding energy between Im_1_ and Im_1_H^+^ is 12.7 kcal mol^−1^, signifying that this pair is the least favored one. In contrast, the HNA1–NA1 distance is elongated from 1.01 Å for the isolated Im_1_ to 1.08 Å for the coordinated structures, which is a more pronounced elongation compared to the other two pairs. This might be attributed to the fact that Im_1_ and Im_1_H^+^ only form one hydrogen bond, while the other two pairs were linked *via* two hydrogen bonds. As a result, the doubly-coordinated pairs are more stable, but the hydrogen bond in the Im_1_/Im_1_H^+^ pair appears to be most pronounced. Since there is no rotational barrier along with a hydrogen bonding interaction, the two Im_1_ fragments align perpendicular to one another, minimizing sterical repulsions (see Fig. 5).

The proton scans for Im_1_/Im_1_H^+^ are the most symmetrical of the three cases presented in this section. This is true when comparing positive and negative *u*_2_ and *u*_3_ values, but also concerning the proton transfer path, *i.e.*, when mirroring along an imaginary line at around *u*_1_ = 1.375 Å. This is reflected in the comparatively small local minima energy difference of 2.1 kcal mol^−1^, which can almost entirely be contributed to conformational relaxations, as both initial and final states are almost identical for intermolecular interactions. The two minima are separated by a barrier of 4.6 kcal mol^−1^ concerning the global minimum.

### 3.2 Onedimensional scans along the reaction coordinate

**Fig. 6:**
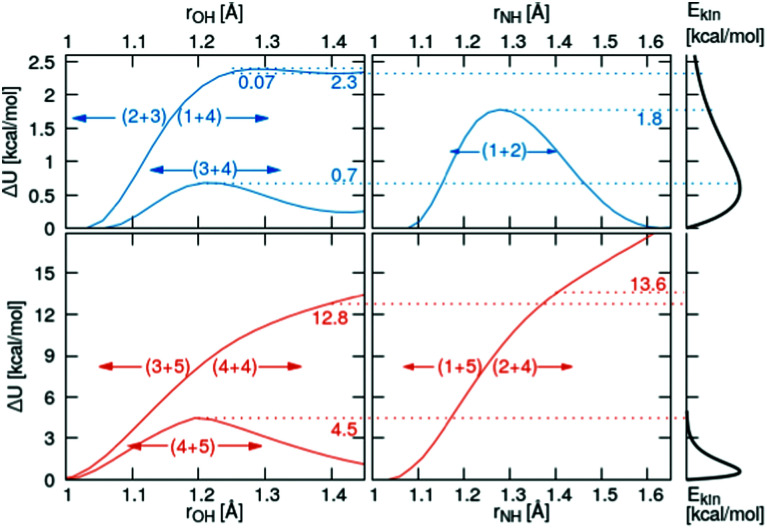
Top row: Quantum-mechanical scans of the reacting pairs in the simple protonation scheme. Bottom row: Additional reactions in the advanced protonation scheme. The right panel concerns the Maxwell–Boltzmann distribution of the kinetic energy.

Relaxed scans along the proton transfer coordinate provide additional insights into the transfer energetics, as nonphysical geometry restraints can be lifted when focusing on only one dimension. All relaxed, onedimensional scans along with an O–H or N–H axis are displayed in Fig. 6. The colored numbers denote the reaction barriers for the reactions in Fig. 3.

The proton transfer between HOAc and Im_1_ is associated with only a minuscule barrier (reactions (1+4) and (2+3)). However, a larger barrier is reported from DFT scans in the study by Kirchner and co-workers^7^ without using a polarizable continuum model. The coordinated structures, where the proton is bound to the Im_1_H^+^, are around 2.3 kcal mol^−1^ less stable than the HOAc/Im_1_ pair. A similar value of 6.9 kJ mol^−1^ = 1.7 kcal mol^−1^ is reported in ref. 8. This energy difference is significantly reduced compared to the 5.4 kcal mol^−1^ in the rigid scans, which is attributed to the added structural flexibility. In this local minimum at *r*_OH_ = 1.41 Å, the NA4–HO1 distance is 1.13 Å, which is >0.1 Å larger than the equilibrium distance in the isolated Im_1_H^+^ ion (1.01 Å), indicating still quite a considerable interaction with the O1 of the acetate.

In comparison, the proton transfer from HOAc to OAc^−^ is significantly more favorable (reaction (3+4)). Here, forward and backward reactions are identical, but the products and educts still are just below 0.2 kcal mol^−1^ different in total energies. This again has to be attributed to the hydrogen bonding interaction between the second O in OAc and the methyl group in HOAc, resulting in different product and educt arrangements as discussed above. With this, the reaction energy is around 2.5 kcal mol^−1^ smaller than that estimated from the rigid scans, which can effectively entirely be attributed to structural relaxations. The reaction barrier between the two local minima is around 0.7 kcal mol^−1^ compared to the global minimum.

Regarding the proton transfer between Im_1_H^+^ and Im_1_ (reaction (1+2)), products and educts are virtually identical in total energies in the relaxed scans. This verifies that the energy difference between the two local minima of 2.1 kcal mol^−1^ in the rigid scans is exclusively attributed to conformational relaxations. Furthermore, the energy barrier is reduced from 4.6 kcal mol^−1^ in the rigid scans to 1.8 kcal mol^−1^ in the relaxed scans.

Three additional pairs were investigated to evaluate the influence of protonated acetic acid (H_2_OAc^+^, 5) in the advanced protonation scheme: H_2_OAc^+^/HOAc, HOAc/Im_1_H^+^, and HOAc/HOAc. From the last two pairs, H_2_OAc^+^ is generated upon proton transfer. Of all pairs investigated in this work, H_2_OAc^+^/HOAc exhibits the largest binding energy at around 20.6 kcal mol^−1^, with HOAc/HOAc and Im_1_H^+^/HOAc binding the weakest, at 10.5 and 9.9 kcal mol^−1^, respectively. Between these three, H_2_OAc^+^/HOAc is the only one with an almost symmetric proton transfer reaction profile (reaction 4+5), as products and reactants are identical in connectivity. The reaction barrier is also moderate compared to the other reaction, including H_2_OAc^+^ at around 4.5 kcal mol^−1^. Still, it is already at least twice that of all proton transfers, which did not include H_2_OAc^+^.

For the two other proton transfers, the formation of H_2_OAc^+^ is very unfavorable. As no meta-stable H_2_OAc^+^ exists according to these scans, the energy at a distance of *r* = 1.4 Å is given. The products H_2_OAc^+^/Im_1_ are 13.6 kcal mol^−1^ less stable than reactants HOAc/Im_1_H^+^ (reactions (1+5) and (2+4)), and H_2_OAc^+^/OAc^−^ is 12.8 kcal mol^−1^ higher in energy compared to the non-charged HOAc/HOAc (reactions (3+5) and (4+4)). Please note that these computations were performed using a polarizable continuum with an experimental dielectric constant of 33.3, which should damp Coulomb interactions between charged molecules by that factor. Additionally, these proton transfers do not traverse any barrier, and the corresponding H_2_OAc^+^ structures do not represent a local minimum. As a result, the reverse transfers (reactions (1+5) and (3+5)) will occur energetically downhill with no activation energy necessary whatsoever.

### 3.3 Reaction probabilities

The onedimensional scans result in reaction barriers depicted in Fig. 6 for the various reactions (*i* + *j*). The transformation of these barriers Δ*U* into reaction probabilities *p*_*ij*_ can be realized *via* various models:

The first model is based on the kinetic energy *E*_kin_ of the protonated molecule. Its normalized Maxwell–Boltzmann distribution8
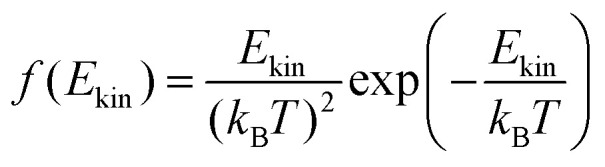
is shown on the right side of Fig. 6. The percentage of molecules with higher kinetic energy than the potential barrier are candidates for the proton transfer. As the distribution in the last equation is normalized, this percentage equals9
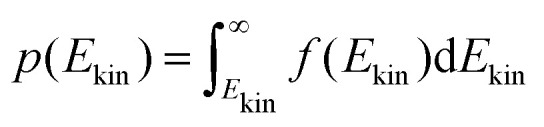
The corresponding values are given in Table 1.

**Table 1:** Basic kinetic parameters to obtain the probability of the investigated reactions. Eqn (11) transforms the rate constants *k* of the collision theory and transition state theory (TST) into probabilities. A time interval of δ*t* = 7 ps has been assumed in Fig. 7

Reaction	Δ*U*_*ij*_	*E*_kin_	TST	Collision
(*i* + *j*)		*p*_*ij*_	*k*_*ij*_	*k*_*ij*_
	[kcal mol^−1^]	[%]	[ps^−1^]	[ps^−1^]
(1 + 4)	2.3	9.8	0.125	0.080
(2 + 3)	0.07	99.4	5.56	3.51
(3 + 4)	0.7	68.4	1.93	1.07
(1 + 2)	1.8	20.1	0.305	0.214
(4 + 4)	>12.8	≪0.1	10^−9^	10^−9^
(3 + 5)		100		
(4 + 5)	4.5	0.5	0.0033	0.0019
(1 + 5)		100		
(2 + 4)	>13.6	≪0.1	10^−9^	10^−10^

The second model is based on transition state theory (TST). The corresponding rate constant *k*_*ij*_ is governed by an Arrhenian factor10
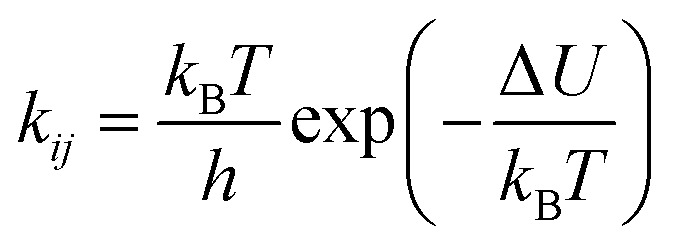
and are also tabulated in Table 1. According to Gillespie^20^ the reaction probabilities *p*_*ij*_ are obtained from the rate constantes *k*_*ij*_ using the concentrations (here mole fractions) *x*_*i*_, *x*_*j*_, and a time interval δ*t* in which the reaction is expected to happen:

11 *p*_*ij*_ = *x*_*i*_·*x*_*j*_·*k*_*ij*_·δ*t*

The third model is based on collision theory.^20^ The reaction rate *k*_*ij*_ is a function of the radius *r*_*i*_ and *r*_*j*_ of the reacting molecules assuming that the reacting molecules are spheres.12
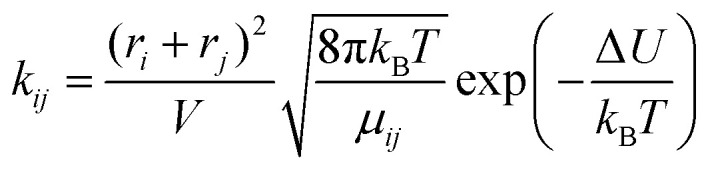
The volume *V* in which the reaction takes place can be estimated *via* the experimental density *ρ*_exp_ and the mole fractions *x*_*i*_ and molar masses *M*_*i*_13
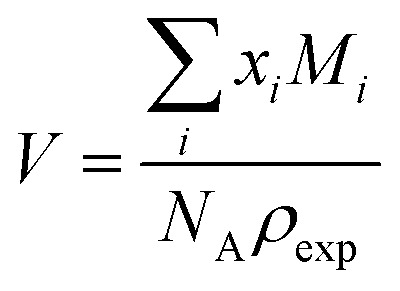
*N*_A_ is the Avogadro constant and *μ*_*ij*_ the reduced mass of the molecules *i* and *j*. The corresponding rate constants in Table 1 are roughly two thirds of the values gained from transition state theory.

**Fig. 7:**
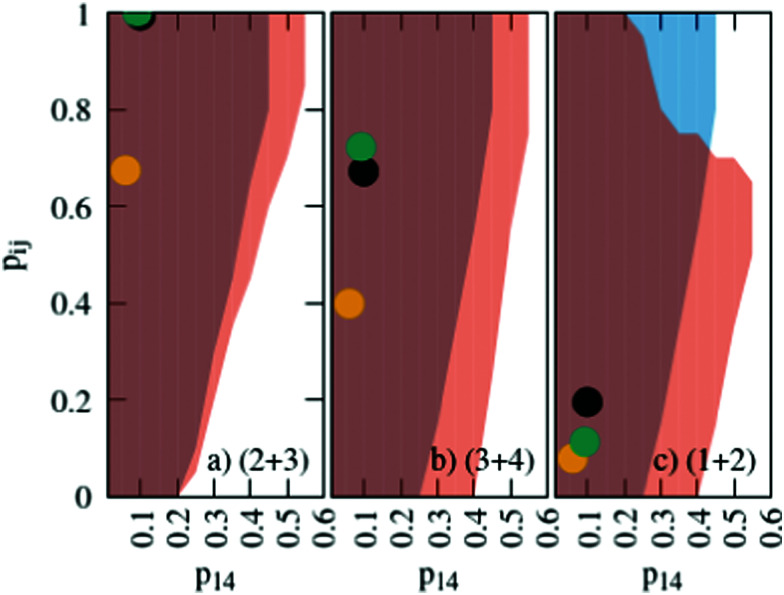
Probabilities for the various reactions in the simple (blue area) and advanced (orange area) protonation scheme as a function of the fundamental reaction (1 + 4) in Fig. 1. The black, green and yellow circles correspond to the kinetic, transition state theory and collision model, respectively. For the latter two, a reaction window of δ*t* = 7 ps is assumed.

The probabilities for the simple reaction scheme for the three models are shown in Fig. 7. A time window of δ*t* = 7 ps in eqn (11) yields probabilities *p*_*ij*_ from the rate constants *k*_*ij*_ which are close to the probability value *p*_*ij*_ from the kinetic energy model. This time interval δ*t* = 7 ps poses an upper limit to some extent as *p*_23_ for TST theory is then already close to unity. However, proton transfer may occur on a much faster time scale. In an AIMD study of Sebastiani *et al.*^6^ protons jumped frequently between carboxylate oxygens and pyridine nitrogens on a sub-picosecond time scale. This may be due to lower potential barriers in their study.

As easily visible from Table 1 all reactions of the advanced protonation scheme with an activation barrier have very low rate constants and consequently negligible probabilities. As a result, there should be no H_2_OAc^+^ present at all. As still some protonated acetic acid were found in AIMD trajectories,^7^ quantum effects like tunneling or many-body effects catalyzing the respective reactions may help to overcome these unfavorable energy pathways. In particular, solvent dipoles may create local electric fields that significantly change the energy surface and, consequently, the proton transfer pathway.^6^ These many-body effects cannot be mapped by the previous section's one- or two-dimensional scans.

### 3.4 Markov chain analysis

Another issue concerns the position of the equilibrium based on the set of distinct probabilities. The values in Table 1 are independent of each other. Hence, these distinct probabilities do not necessarily lead to the mole fractions found in the experiment.

We performed a systematic scan of all reaction probabilities *p*_*ij*_ in Fig. 4 using the Markov chain model in steps of 0.05 and checked for the final concentrations 
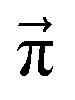
 as well as the charge neutrality. In order to save computational time, the probabilities of reaction (4+4) and (2+4) were restricted to an upper limit of 20%. The lower limit of the probabilities for reaction (3+5) and (1+5) was set to 80%. These values are well beyond those expected by the reaction barriers of 12.8 kcal mol^−1^ and 13.6 kcal mol^−1^. Nevertheless, more than 800 million sets of probabilities were scanned in the advanced protonation scheme based on these limits.

The systematic scans for the simple and advanced protonation scheme were performed separately, and the results are visualized in Fig. 7 as blue (simple) and orange (advanced) areas. Given a particular probability of *p*_14_ all *p*_*ij*_ within the shaded areas yield the correct experimental mole fractions. The shaded areas of both protonation schemes overlap to a vast extent. However, the advanced protonation scheme allows for slightly higher *p*_14_ probabilities. Fortunately, all probabilities calculated in the previous section for the kinetic, collision, and transition state models are covered by the simple and advanced Markov chains in Fig. 4. The unlikely reactions (4+4) and (2+4) as well as the automatic reactions (3+5) and (1+5) of the advanced protonation scheme (see Fig. 3) do not restrict the findings in Fig. 7 as for each *p*_14_ all tested probabilities *p*_*ij*_ of these reactions lead to valid mole fractions 
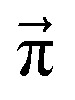
 using eqn (6).

**Fig. 8:**
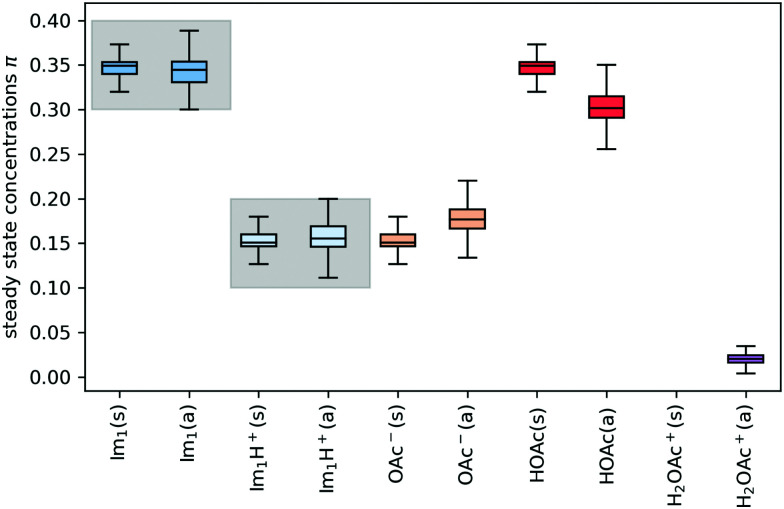
Steady state concentrations (= mole fractions) of the compounds in the simple (s) and advanced (a) protonation scheme. The average final concentrations 
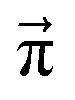
 are {0.349,0.151,0.151,0.349,0.000} for the simple protonation scheme and {0.345,0.155,0.178,0.302,0.020} for the advanced protonation scheme.

In addition to the valid probabilities *p*_*ij*_, box plots of the resulting steady state mole fractions 
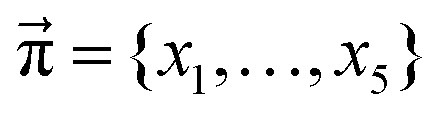
 are obtained in Fig. 8 for all scanned probability sets which fulfill charge neutrality, balance of species and the experimental ratio of ionic:neutral molecules. In the simple protonation scheme, the ratio of 0.349 : 0.151 corresponds to roughly 70% neutral/30% ionic molecules and is in the center of gray shaded areas, which indicates our limits for the imidazolium-based species. The box plots of the mole fractions shift in the advanced protonation scheme as the increased acetate concentration can be compensated by an increased concentration of protonated acetic acid to keep charge neutrality. However, the mole fraction *x*_5_ = 0.02 of H_2_OAc^+^ is relatively low due to the unfavorable energy state. Applying the probabilities for the kinetic energy model (black circles in Fig. 7) or the TST (green circles) and collision theory (yellow circles) using δ*t* = 7 ps result in *x*_5_ < 10^−5^. The values *x*_5_ > 0.01 are only reached for high values of *p*_44_ and *p*_24_ of the unfavorable reactions in the rigid scan. The increased concentrations of OAc^−^ and H_2_OAc^+^ decrease the concentration of HOAc because the sum *x*_3_ + *x*_4_ + *x*_5_ of carboxylate based species should yield *x*_1_ + *x*_2_ of the imidazolium based species.

## 4 Concluding discussion

The present work combines quantum-mechanical with statistical mechanics calculations to investigate the equilibrium between 1-methylimidazole + acetic acid and 1-methylimidazolium acetate. This equilibrium is accompanied by several other (de-)protonation reactions. Twodimensional quantum-mechanical scans show that the proton transfer most probably happens *via* a direct transfer between the active oxygens and/or nitrogens. Thus, this distance may be used as a reaction coordinate for onedimensional scans showing various energy barriers for the investigated reactions.

These reactions may be decomposed in a simple and advanced reaction scheme: The simple reaction scheme contains four reactions between 1-methylimidazole, 1-methylimidazolium, acetate, and acetic acid. The advanced reaction scheme augments this set of reactions and includes the formation and deprotonation of the protonated acetic acid. Distinct probabilities can be determined based on a kinetic model, transition state theory, and collision theory using reaction windows of 7 ps for the latter two models. Markov chain models for the simple and advanced protonation scheme demonstrate that these probabilities lead to the experimental composition of the involved species.

As the predicted probabilities for the reactions involving the protonated acetic acid are practically zero, artificial probabilities will be used to investigate the effect of this species for the charge transport in the polarizable molecular dynamics simulations. Please keep in mind that the protonated acetic acid is the only molecule that qualifies for a direct Grotthus mechanism for charge transport. The protonation of 1-methylimidazole only yields an indirect Grotthus mechanism, then molecular rotation and subsequent deprotonation of the 1-methylimidazolium.

## Conflicts of interest

There are no conflicts to declare.

## Supplementary Material
